# Factors Affecting Infant Feeding Practices Among Women With Severe Mental Illness

**DOI:** 10.3389/fgwh.2021.624485

**Published:** 2021-04-09

**Authors:** Natasha Baker, Laura Potts, Stacey Jennings, Kylee Trevillion, Louise M. Howard

**Affiliations:** ^1^Health Services and Population Research, Institute of Psychiatry, Psychology and Neuroscience King's College London, London, United Kingdom; ^2^Centre for Psychiatry, Wolfson Institute of Preventive Medicine, Queen Mary University London, London, United Kingdom

**Keywords:** infant feeding, breast feeding, perinatal, postpartum, severe mental illness, mixed methods

## Abstract

**Background:** The health benefits of breastfeeding are well-established but for mothers with severe mental illness (SMI), the decision to breastfeed can be complex. Very few prior studies have investigated the infant feeding choices of women with SMI, or the factors associated with this. Our aims were to examine antenatal infant feeding intentions and infant feeding outcomes in a cohort of women admitted for acute psychiatric care in the first postpartum year. We also aimed to examine whether demographic and clinical characteristics associated with breastfeeding were similar to those found in previous studies in the general population, including age, employment, education, BMI, mode of delivery, smoking status, and social support.

**Methods:** This study was a mixed-methods secondary analysis of a national cohort study, ESMI-MBU (Examining the effectiveness and cost-effectiveness of perinatal mental health services). Participants had been admitted to acute care with SMI in the first postpartum year. Infant feeding outcomes were retrospectively self-reported by women during a 1-month post-discharge interview. Free-text responses to questions relating to infant feeding and experience of psychiatric services were also explored using thematic analysis.

**Results:** 144 (66.1%) of 218 women reported breastfeeding (mix feeding and exclusive breastfeeding). Eighty five percentage of the cohort had intended to breastfeed and of these, 76.5% did so. Factors associated with breastfeeding included infant feeding intentions, employment and non-Caucasian ethnicity. Although very few women were taking psychotropic medication contraindicated for breastfeeding, over a quarter (*n* = 57, 26.15%) reported being advised against breastfeeding because of their medication. Women were given this advice by psychiatry practitioners (40% *n* = 22), maternity practitioners (32.73% *n* = 18) and postnatal primary care (27.27% *n* = 15). Most women stopped breastfeeding earlier than they had planned to as a result (81.1% *n* = 43). Twenty five women provided free text responses, most felt unsupported with infant feeding due to inconsistent information about medication when breastfeeding and that breastfeeding intentions were de-prioritized for mental health care.

**Conclusion:** Women with SMI intend to breastfeed and for the majority, this intention is fulfilled. Contradictory and insufficient advice relating to breastfeeding and psychotropic medication indicates that further training is required for professionals caring for women at risk of perinatal SMI about how to manage infant feeding in this population. Further research is required to develop a more in-depth understanding of the unique infant feeding support needs of women with perinatal SMI.

## Introduction

The benefits of breastfeeding to the physical and psychological well-being of mothers and their babies are well established (1, 2). Breastfeeding initiation in England is around 74% (3) but falls to around 34% by 6 months (4). Breastfeeding initiation and duration in the general population are predicted by demographic factors (maternal age, education, income, employment and smoking status), clinical characteristics (body mass index, parity and gestational age), social and professional support (5). There is also now growing evidence supporting the relationship between breastfeeding outcomes and maternal mental health (6–8). Whilst much of the literature remains contradictory, it is generally accepted that positive breastfeeding experiences can be protective of common mental health conditions like depression and anxiety disorders, but negative experiences and early cessation of breastfeeding can lead to poor mental health (8). Those with existing depressive symptoms also report greater breastfeeding difficulties and reduced breastfeeding self-efficacy than women without depression (9). The importance of antenatal infant feeding intentions has also recently become acknowledged, and breastfeeding has been described as a moderator, decreasing or increasing anxiety and depressive symptoms depending on the infant feeding intentions of the mother (8, 10, 11). Despite the well-established evidence base surrounding infant feeding and common mental health conditions, very little is currently known about the infant feeding intentions and practices of women with the most severe mental illnesses (SMI).

Perinatal SMI include conditions such as psychosis, schizophrenia, bipolar disorder and severe depression. It affects those with pre-existing psychiatric conditions who become pregnant, accounting for around 2.8% of women in the general population, and those who develop a severe post-partum psychiatric illness as their first episode, affecting 1–2 per 1,000 births (12). Motherhood is central to women with SMI (13), as it is for other women but their experiences are often complicated by their mental health. Pregnancy and the transition to motherhood for women with SMI can be particularly challenging and maternal suicide remains the leading cause of direct maternal death within the first year after birth (14).

Whilst breastfeeding has been described by women with SMI as a defining moment of motherhood, it is also reported to be one of the most challenging (15). There are several potential reasons why breastfeeding could be particularly problematic for women with SMI. The use of psychotropic medication and some symptoms of psychiatric illness can make women drowsy and unresponsive to infant feeding cues, whilst sleep deprivation and stress can exacerbate existing illnesses like bipolar disorder (16, 17).

Although very few psychotropic medications are completely contraindicated for breastfeeding, stopping medication is common (18) and conflicting advice from health care professionals can make women more vulnerable to de-prescribing and psychological deterioration (19). These factors make infant feeding decisions for women with SMI and their families more complex. A qualitative study exploring how women with bipolar disorder make decisions about pregnancy reported some incidental findings relating to infant feeding. Several women in this study decided against breastfeeding antenatally due to psychotropic medications they were taking (namely Lithium) and concern for postpartum relapse. However, others reported stopping their medication because of the pressure they felt to breastfeed, or that stigma and guilt relating to not breastfeeding led to inadequate formula feeding support from maternity staff and worry about being judged by other mothers (13).

There are significant benefits of breastfeeding for mothers with SMI, including the potential for improved maternal confidence, self-efficacy and mother-baby attachment (20–22). However, to our knowledge no prior study has investigated factors influencing women's infant feeding choices when they have SMI. This study aims to examine the infant feeding intentions and outcomes of a cohort of women with both enduring SMI and acute onset perinatal SMI in the first postnatal year, and some of the biopsychosocial factors associated with this. This study also explores some of the qualitative factors relating to infant feeding for women with SMI by observing free text responses.

## Methods

### Sample

This was a mixed-methods secondary data analysis of the ESMI (Effectiveness of perinatal mental health services) study. ESMI was a quasi-experimental cohort study embedded within existing service matrices, examining the effectiveness and cost effectiveness of psychiatric mother and baby units (MBU), compared with general psychiatric inpatient wards and crisis resolution teams (CRT) for mothers with psychiatric illness in the first postpartum year (23). The study obtained NHS Research Ethics Committee approval from the London-Camber well St. Giles committee (number: 14/LO/0765) and this secondary data analysis was conducted in accordance with the Declaration of Helsinki and General Data Protection Regulations.

The ESMI cohort included 279 women, recruited from 42 mental health service provider organizations across England and Wales between 2015 and 2018. Women were eligible if they had a psychiatric disorder and required acute care from at least one psychiatric service (MBUs, CRTs, generic acute wards), or any combination of all three, during the first year after childbirth and had capacity to consent at the point of or after discharge. This includes women with anxiety disorders and personality disorders who had been admitted under acute care and fulfilled inclusion criteria. Women were not included if they were under acute care prophylactically or if they had had their baby permanently removed prior to admission. Short- and long-term follow-up assessments were undertaken at one- and 12-months post-discharge from psychiatric services. Detailed description of recruitment methods and study procedures can be found in the study protocol (23).

The sample size for this secondary data analysis was derived of 218 women from the cohort who answered questions about their antenatal infant feeding intentions and actual infant feeding outcomes. Twenty five women from the cohort also gave more in-depth details about their experiences surrounding infant feeding within free-text responses. This data was used to conduct a qualitative thematic analysis of factors influencing breastfeeding intention and outcomes in women with perinatal SMI.

### Measures

#### Breastfeeding

Women retrospectively self-reported their antenatal infant feeding intentions and how they had actually fed their babies in an Obstetric History Questionnaire (OHQ), during a structured research interview at 1-month following discharge from psychiatric services. Responses included either *breast, mixed or formula feeding*. The primary outcome was any breastfeeding reported (yes/no). For the purpose of this study, breastfeeding is referred to as breastfeeding in any capacity and women were dichotomously categorized as either breastfeeding (exclusively breastfeeding, supplementing some feeds with formula milk, or giving expressed breast milk via bottle) or no breastfeeding (formula feeding only). Participants were not specifically asked within the primary study (which focused primarily on effectiveness of services) when they had initiated breastfeeding or how long they breastfed for. Breastfeeding initiation could have occurred before the onset of the illness, and could have contributed to acute care.

Mediating factors including psychotropic medication use and advice against breastfeeding because of medication. Participants were asked if they had been advised to stop breastfeeding due to psychotropic medication they were taking, and if so, which medication this was, who provided this advice *(psychiatry, midwifery, primary care practitioners*), and their subsequent actions (*stopped breastfeeding, continues or mix fed*). Participants were then invited to provide further detail on their experience in a free-text response. As part of the structured research interview, participants were also asked to comment on experiences of psychiatric services within the Perinatal Voice Questionnaire (PVQ), a 27-item self-report questionnaire. Free-text responses from the PVQ and OHQ were analyzed and themes were developed.

#### Clinical Characteristics

Psychiatric characteristics were collected retrospectively from clinical case notes and retrospectively self-reported by women during the one-moth post discharge research interview. These include ICD-10 psychiatric diagnosis (24) recorded for the admission; Depressive disorder (F32–39), Bipolar disorder (F31), Schizophrenia (F20–29), anxiety and adjustment disorders (F40–48) and personality disorders (F60–69). Some women had also been coded as disorders associated with the postpartum (not elsewhere classified) (F53) and we acknowledge the limitations of this diagnosis as it tells us very little about the illness itself; Previous episodes of psychiatric illness (binary yes/no), and onset of the current episode in relation to the delivery (prior to pregnancy, pregnancy, postpartum).

#### Covariates

Other demographic and clinical characteristics analyzed included; age (16–24, 25–34, 35–49), ethnicity (binary; Caucasian/non-Caucasian. Cell frequency for white other, black African, black Caribbean, Asian, mixed and other was <5), highest level of education (school leaver & below, higher education, degree and above), household income (<£31,000, £31,000-£61,000, £61,000+), partner status (binary; single/partnered), employment status prior to pregnancy (binary; employed/un-employed), self-reported smoking status during pregnancy (binary; smoker/non-smoker), substance abuse (binary; Yes/No), body mass index (healthy, overweight, obese), previous live births (binary; Yes/No), gestational age at birth [binary; term(37–42 weeks)/pre-term (32–36 weeks)], mode of birth [spontaneous vaginal birth, assisted vaginal birth, cesarean section (emergency and planned)], infants birth weight [low birth weight (<2500g), normal (2500–4000g), macrosomia (>4000g)] and pregnancy planning (binary; planned/un-planned).

### Data Analysis

Quantitative data were analyzed using STATA version 16 (StataCorp, 2019). Women's baseline socio-demographic and clinical characteristics were described using summary statistics (*N*/%) by breastfeeding and non-breastfeeding groups. Univariate analysis was performed to establish those characteristics that were significantly associated with breastfeeding (*p* < 0.05), and odds ratios were presented from these analyses. These initial predictors were then included in a multivariate regression model, together with partner status, gestational age at birth and parity (previous children), which are factors known to be associated with breastfeeding from current literature in the general population (5, 25, 26). Breastfeeding intentions were not included in the multivariate model as univariate analysis demonstrates almost perfect prediction. Variables with >20 missing observations (income and BMI) were also not included in the multivariate model as missing observations resulted in a 20% loss of cases in the complete case analysis. However, a sensitivity analysis to impute these variables and hence include in the model (if significant) was performed to check for any potential bias this may have caused. The multivariate logistic regression model from the primary analysis was used but also including variables BMI and income which were imputed using multiple imputation. The multiple imputation model included the full set of variables used in the primary analysis.

A complete case analysis on the primary outcome was carried out using multivariate logistic regression and the binary variable breastfed (yes/no) was used as the dependent variable. Demographic factors found to be associated with breastfeeding in the general population were adjusted for in the model as a priori confounders, including maternal age, ethnicity and education (4).

A separate multivariate logistic regression was carried out to examine associations with the primary outcome only in women who had antenatal intentions to breastfeed. Adjusted odds ratios (ORs) and 95% confidence intervals (CIs) are presented for each independent variable. Model fit was assessed using the likelihood ratio and deviance residuals.

Qualitative free-text responses from the PVQ and OHQ were transcribed verbatim and analyzed according to Braun and Clarke's thematic analysis (TA) (27). Transcripts were read several times to familiarize the researchers with the data, line-by-line coding was carried out within each transcript. Initial codes were developed and allocated to text sections, which were then developed into themes across topics. Themes were reviewed against codes and the dataset as a whole by NB and SJ.

## Results

### Sample Characteristics

218 women answered questions about their infant feeding intentions and outcomes. Background demographic and clinical characteristics of the sample are presented in Table 1. The majority of participants were aged between 25 and 34 years (54.6%), were Caucasian (77.1%) and had some level of postsecondary education (70.2%). Over half of the sample were living on below the national average household income [<£31,000 Per Annum (28)] (56.8%) and most were in a significant relationship at the point of pregnancy (93.6%), though this fell to 82.6% after pregnancy. Fifty three point two percentage were having their first baby and for the majority (82.5%), the onset of psychiatric illness had occurred either in pregnancy (28.6%) or postnatally (53.9%), although many reported experiencing previous episodes in the 2 years prior to the index admission (70.5%).

**Table 1:** Socio demographic and Clinical Characteristics of women who breastfed vs. women who did not breastfeed (*n* = 218).

**Variable**	**Breastfed-Yes**	**Breastfed-No**		
**Age (*n* = 218)**	***N* (%)**	***N* (%)**	**OR (95% CI)**	***P*-Value**
16–24	19 (57.6%)	14 (42.4%)	*Ref*	
25–34	85 (71.4%)	34 (28.6%)	1.8 (0.8–4.1)	0.13
35–49	40 (60.6%)	26 (39.4%)	1.1 (0.5–2.6)	0.77
**Ethnicity (*****n*** **=** **218)**				
Caucasian	105 (62.5%)	63 (37.5%)	*Ref*	
Non-Caucasian	39 (78%)	11 (22%)	2.1 (1.0–4.5)	0.05
**Education (*****n*** **= 218)**				
School leaver & below	22 (57.9%)	16 (42.1%)	*Ref*	
Higher education	100 (65.4%)	53 (34.6%)	1.4 (0.7–2.8)	0.39
Degree & above	22 (81.8%)	5 (18.5%)	3.2 (1.0–10.3)	0.05
**Household income (*****n*** **= 197)**				
Low (£0–30,999)	70 (62.5%)	42 (37.5%)	*Ref*	
Middle (£31,000–60,999)	32 (65.3%)	17 (34.7%)	1.1 (0.6–2.3)	0.73
High (>£61,000)	30 (83.3%)	6 (16.7%)	3.0 (1.2–7.8)	0.02
**Employment Status (*****n*** **= 218)**				
Employed	106 (73.6%)	43 (58.1%)	2.0 (1.1–3.6)	0.02
**Partner status (pregnancy) (*****n*** **= 218)**				
Partner/married	138 (95.8%)	66 (89.2%)	2.8 (0.9–8.4)	0.07
**Partner status (after pregnancy) (*****n*** **= 218)**				
Partner/married	123 (85.4%)	57 (77.03%)	1.7 (0.9–3.6)	0.13
**Smoking status (*****n*** **= 218)**				
Smoker	24 (16.7%)	23 (31.1%)	0.4 (0.2–0.9)	0.02
**Substance Misuse (*****n*** **= 218)**				
Yes	10 (70.4%)	4 (28.6%)	1.3 (0.4–4.3)	0.66
**BMI (*****n*** **= 176)**				
Healthy	55 (80.9%)	13 (19.12%)	*Ref*	
Overweight	28 (51.8%)	26 (48.15%)	0.3 (0.1–0.6)	<0.01
Obese	34 (63%)	20 (37%)	0.4 (0.2–0.9)	0.03
**Intended to Breastfeed (*****n*** **= 218)**				
Yes	142 (98.6%)	44 (59.4%)	48 (11.1–211)	<0.01
**Parity (previous live births) (*****n*** **= 218)**				
Yes	64 (44.4%)	38 (51.3%)	0.8 (0.4–1.3)	0.33
**Gestation at birth (*****n*** **= 199)**				
Preterm (32–36 weeks)	14 (10.5%)	13 (19.7%)	0.5 (0.2–1.1)	0.08
**Birth Weight (*****n*** **= 196)**				
<2500g	8 (6.2%)	7 (10.4%)	*Ref*	
2500–4000g	113 (87.6%)	55 (82.1%)	1.8 (0.6–5.2)	0.28
>4000g	8 (6.2%)	5 (7.5%)	1.4 (0.3–6.3)	0.66
**Mode of birth (*****n*** **= 211)**				
Spontaneous vaginal birth (SVB)	74 (63.25%)	43 (36.75%)	*Ref*	
Assisted	18 (52.94%)	16 (47.06%)	0.7 (0.3–1.4)	0.28
Cesarean birth	47 (78.33%)	13 (21.67%)	2.1 (1.0–4.3)	0.04
**Pregnancy planning** ***(*****n*** **= 218)***				
Unplanned	52 (36.1%)	38 (51.3%)	0.5 (0.3–0.9)	0.03
**Onset of Psychiatric disorder (*****n*** **= 217)**				
Prior to pregnancy	19 (50%)	19 (50%)	*Ref*	
Pregnancy	41 (66.13%)	21 (33.87%)	2.0 (0.9–4.5)	0.11
Postpartum	83 (70.94%)	34 (29.06%)	2.4 (1.2–5.2)	0.02
**Previous episodes (subsequent 2yrs) (*****n*** **= 217)**				
No	44 (30.8%)	20 (27%)	1.2 (0.6–2.2)	0.57
**Index Psychiatric Diagnosis (*****n*** **= 213)**				
Depression/unipolar disorder	62 (72.9%)	23 (27.1%)	*Ref*	
Bipolar Disorder	35 (66%)	18 (34%)	0.7 (0.3–1.5)	0.39
Schizophrenia	8 (57.1%)	6 (42.9%)	0.5 (0.2–1.6)	0.24
Anxiety Disorder	22 (71%)	9 (29%)	0.9 (0.4-2.3)	0.83
Disorders associated with the puerperium (not elsewhere classified)	7 (58.3%)	5 (41.7%)	0.5 (0.1–1.8)	0.30
Personality Disorder	7 (38.9%)	11 (61.1%)	0.2 (0.1–0.7)	<0.01

Univariate regression analysis demonstrated some evidence that education (Degree & above*:* OR 3.2, 95% CI:1.0–10.3), household annual income (>£61,000: OR 3.0, CI: 1.2–7.8), employment status (Employed: OR 2.0, CI: 1.1–3.6), BMI (Overweight: OR 0.3 CI: 0.1–0.6), smoking status (Smoker: OR 0.4 CI: 0.2–0.9) and ethnicity [Non-Caucasian: (women from Black, Asian and other ethnic minority backgrounds) OR 2.1 CI: 1.0–4.5] were associated with breastfeeding outcomes. As were unplanned pregnancy (OR 0.5, CI: 0.3–0.9), cesarean birth (OR 2.1, CI: 1.0–4.3), postpartum psychiatric onset (OR 2.4, CI: 1.2–5.2) and personality disorder (OR 0.2, CI: 0.1–0.7). These variables were therefore included in the multivariate model. BMI and household income were found to be associated with breastfeeding from the univariate analysis but were not included in the multivariate complete case analysis due to 20% missing data in these variables.

### Infant Feeding Intentions and Outcomes

144 (66.1%) women reported breastfeeding in some capacity (42.2% breastfed exclusively, 23.8% mix fed) and 74 (33.9%) formula fed their babies. 186 (85.3%) women had antenatal intentions to breastfeed and these women were significantly more likely to breastfeed than those who had not intended to breastfeed (OR 48.0, 95% CI: 11.1–211.0, *p* < 0.01). 76.3% of the women intending to breastfeed reported doing so and exclusive breastfeeding was also higher in this group (48.7% breastfed exclusively, 27.8% mix fed).

Over a quarter (*n* = 57, 26.4%) reported being advised to stop breastfeeding because of psychotropic medications they were taking, and the majority of these women stopped breastfeeding earlier than they had planned to (81.1% *n* = 43). Women were given this advice mostly by acute psychiatry services (40% *n* = 22). However, 32.7% (*n* = 18) were told to stop by maternity services and 27.3% (*n* = 15) were advised by postnatal universal services, such as health visitors, community midwives and GPs. Women advised against breastfeeding were mostly medicated with an atypical antipsychotic medication (e.g., Quetiapine and Olanzapine) and/or a selective serotonin reuptake inhibitor (SSRI) (e.g., Sertraline, citalopram and Fluoxetine). Only one participant reported taking Lithium, which is not generally used during lactation (29) and polypharmacy was present in one participant who formula fed, as she had intended. Findings from free-text comments explore key issues surrounding misplaced and contradictory advice about breastfeeding and medication in more detail.

### Multi-Variable Analysis

Findings from the complete case multivariate logistic regressions analysis are presented in Table 2. In the first analysis of 188 women, after adjustment for *a priori* confounders, there was weaker evidence for personality disorder and cesarean birth being associated with breastfeeding (AOR 0.3 CI: 0.08–1.1; AOR 2.2 CI: 0.9–5.3, respectively) but there was stronger evidence for associations with non-Caucasian ethnicity (AOR 3.1 CI:1.2–8.1) and employment (AOR 2.5 CI:1.1–5.8).

**Table 2:** Characteristics associated with breastfeeding in women with SMI.

	**Complete case analysis of cohort (*n* = 188)**		**Women with antenatal intention to breastfeed (*N* = 163)**	
**Variable**	**AOR (95% CI)**	***P*-value**	**AOR (95% CI)**	***P*-value**
**Relationship Status (pregnancy)**				
Partner/Married	3.6 (0.8–15)	0.08	4.6 (0.6–33.7)	0.13
**Gestation at birth**				
Preterm (32–37 weeks)	0.5 (0.2–1.3)	0.17	0.5 (0.2–1.8)	0.31
**Pregnancy planning**				
Unplanned	0.7 (0.3–1.5)	0.33	0.7 (0.3–1.7)	0.38
**Mode of birth**				
Assisted vaginal	0.8 (0.3–2.2)	0.65	0.9 (0.3–2.8)	0.79
Cesarean section	2.2 (0.9–5.3)	0.07	3.1 (0.99–9.5)	0.05
**Psychiatric Onset**				
Pregnancy	2.1 (0.7–6)	0.18	2.1 (0.6–7.9)	0.27
Postpartum	1.5 (0.5–4.1)	0.42	1.8 (0.5–6.7)	0.39
**Psyc Diagnosis (for index admission)**				
Bipolar Disorder	0.5 (0.2–1.3)	0.16	0.4 (0.1–1.03)	0.06
Schizophrenia	1.2 (0.3–5.7)	0.78	4.3 (0.4–46.9)	0.23
Anxiety disorders	0.8 (0.3-2.4)	0.72	0.9 (0.2-3.1)	0.85
Disorders associated with the puerperium (not elsewhere classified)	0.5 (0.1–2.1)	0.33	0.3 (0.05–1.6)	0.15
Personality Disorder	0.3 (0.08–1.1)	0.07	0.8 (0.1–5.2)	0.79
**Ethnicity**				
Non-Caucasian	3.1 (1.2–8.1)	0.02	4.0 (1.1–14.3)	0.03
**Age**				
25–34	2.2 (0.8–6.3)	0.13	3.4 (0.9–11.7)	0.06
35–49	0.9 (0.3–3)	0.91	1.4 (0.3–5.7)	0.65
**Education**				
Post-secondary education	0.4 (0.1–1.2)	0.09	0.2 (0.03–0.8)	0.03
Degree and above	0.8 (0.2–3.7)	0.75	0.3 (0.03–2.5)	0.25
**Employment prior to pregnancy**				
Working	2.5 (1.1–5.8)	0.03	2.5 (0.8–7.3)	0.10
**Smoking status (during pregnancy)**				
Smoker	0.8 (0.3–2)	0.59	0.5 (0.1–1.5)	0.21
**Previous live births**				
Yes	0.7 (0.3–1.5)	0.34	1.2 (0.5–3.4)	0.67

A further complete case analysis (*n* = 163) was carried out in women who had intended to breastfeed antenatally and demonstrated that women from non-Caucasian ethnicities were four times more likely to breastfeed than Caucasian women (AOR 4.0 CI: 1.1–14.3) but there was weaker evidence for an association with employment status. The sensitivity analyses on the *n* = 188 and *n* = 163 women including variables BMI and income imputed to a full dataset confirmed there was no change in the results (Sensitivity analysis can be found in Appendix A).

Our final models have likelihood ratio statistics of 37.9 (*p* = 0.009) and 34.6 (*p* = 0.02), respectively, indicating a good model fit. A plot of the deviance residuals against the fitted values showed no striking trend to indicate any problems in the models.

### Overview of Free-Text Findings

25 women provided free -text responses, of whom 15 (60%) breastfed. Background demographic and clinical characteristics of the qualitative sample are presented in Table 3. Sample characteristics were similar to the quantitative cohort in age [60% (25–34 years)], ethnicity (76% Caucasian) and partner status (96% in a relationship). However, women providing free-text responses reported higher educational qualifications (52% had a degree or higher) and a higher percentage were having their first baby (*n* = 17, 71%). Sixty three percentage had been advised to stop breastfeeding due to medication use, opposed to 26.4% of the quantitative sample. Two overarching themes were identified; “unsupportive and inconsistent” and “de-Prioritization of breastfeeding intentions.” Participant's quotes are described under an anonymised ID.

**Table 3:** Socio demographic and clinical characteristics of qualitative sample (*n* = 25).

**Variable**	***N* (%)**
**Age**	
16–24	1 (4%)
25–34	15 (60%)
35–49	9 (36%)
**Ethnicity**	
Caucasian	19 (76%)
Non-Caucasian	6 (24%)
**Education**	
School leaver & below	2 (8%)
Post-secondary qualification	10 (40%)
Degree & above	13 (52%)
**Household income**	
Low (£0–30,999)	11 (44%)
Middle (£31,000–60,999)	10 (40%)
High (>£61,000)	4 (16%)
**Employment Status**	
Employed	18 (72%)
**Partner status (pregnancy)**	
Partner/married	24 (96%)
**Partner status (after pregnancy)**	
Partner/married	20 (80%)
**Smoking status (during pregnancy)**	
Smoker	2 (8%)
**BMI**	
Healthy	9 (41%)
Overweight	7 (32%)
Obese	6 (27%)
**Previous live births**	
No	17 (71%)
**Gestation at birth**	
Term (37–42 weeks)	20 (87%)
**Birth Weight**	
<2500g	3 (13%)
2500–4000g	19 (83%)
>4000g	1 (4%)
**Mode of birth**	
Normal vaginal birth (NVB)	11 (48%)
Assisted	4 (17%)
Cesarean birth	8 (35%)
**Pregnancy planning**	
Planned	18 (72%)
**Onset of Psychiatric disorder**	
Prior to pregnancy	4 (16%)
Pregnancy	6 (24%)
Postpartum	15 (60%)
**Previous episodes (subsequent 2yrs)**	
Yes	16 (64%)
**Index Psychiatric Diagnosis**	
Depression/unipolar disorder	10 (40%)
Bipolar disorder	5 (20%)
Anxiety & adjustment disorders	6 (24%)
Disorders associated with the puerperium NEC	3 (12%)
Personality Disorder	1 (4%)
**Breastfed**	
Yes	15 (60%)
**Advised to stop BF due to psychotropic medication**	
Yes	15 (63%)

#### Unsupportive and Inconsistent

The free-text comments confirm that breastfeeding choices for women with SMI can be very complex and multifaceted, particularly in regard to medication. Health care providers are described as compounding these challenges further and many women felt unsupported or that they had received insufficient information and advice from health professionals about medication.

“*No-one explained meds and what it was for. It was particularly hard given I had to stop breastfeeding” (117)*.

“*I wish they had told me to stop breastfeeding rather than give me diluted medication” (110)*.

One participant described being told to make a binary choice “I was told to stop feeding or stop meds” by an “uninformed health visitor who had no idea about psych meds and breastfeeding” (118). Women reported being given contradictory information about breastfeeding whilst taking psychotropic medication from different health professionals during pregnancy and the postpartum, in one case from the same individual at different timepoints.

*Early in pregnancy, the mental health midwife said not to take fluoxetine if breastfeeding and to change to sertraline or citalopram. Next time I saw her later on and she said I could stay on fluoxetine if I was happy on it (114)*

“*Was on Quetiapine in pregnancy. Psychiatrist said she could not breastfeed on the medication but OB/GYN said I could” (123)*.

One participant discussed how incorrect and contradictory advice prevented her from breastfeeding, despite it being safe to do so.

“*Under HTT (home treatment team) was given olanzapine plus told not to breastfeed as it could kill the child and some info given to husband. Did ask for a breastfeed-friendly anti-depressant but was not given them. Following convo (conversation) with perinatal psychiatrist who said I could have breastfed on this medication but my milk production had stopped- very unhappy about this” (124)*.

Women described this as confusing and distressing, resulting in a lack of confidence in health professional and prompting some women to conduct their own research or to disregard medical advice.

“*Carried on feeding baby” (118*).

“*Breastfed anyway” (116*).

Stopping breastfeeding due to medication had a significant impact on some women, who felt that services did not fully consider or support for this transition.

“*I had to go on olanzapine which meant I had to stop breastfeeding- this had a massive negative impact” (111)*.

#### De-Prioritization of Breastfeeding Intentions

Decisions about breastfeeding and fulfilling breastfeeding intentions evoked strong feelings for participants. Women felt that due to the complexities of their mental health, breastfeeding was not considered relevant and was “de-prioritized” for other aspects of acute care.

“*The service not at all focused on my desire to continue breastfeeding. My willingness to comply but have my right to breastfeed supported was not prioritized” (124)*.

Despite many mothers expressing strong preferences to continue breastfeeding, they often felt that their preferences were ignored.

“*Medication was an issue as I was initially given medication that specified it should not be taken while breastfeeding, when I had made my wish to breastfeed very clear” (109)*.

Others felt that because of this, the responsibility to initiate discussions about feeding fell to them

“*I had to raise concerns with medication and breastfeeding” (118)*.

Some women identified particular points within the breastfeeding journey where support was lacking, such as restarting breastfeeding after cessation during admission. For women who stopped breastfeeding during admissions, practical facilities were often absent or inadequate which contributed to discomfort and embarrassment. Whilst those who continued breastfeeding described a lack of privacy and discomfort.

“*It was painful to stop breastfeeding. I had to hand express into a vomit bowl” (117)*.

“*For me the situation in the hospital wasn't good, because in that moment I wanted to stay in my house with my husband and my mum. Sometimes when I was breastfeeding my baby, personal team arrive in my bedroom and I felt uncomfortable with that situation, because I needed peace for breastfeeding my baby” (107)*.

The significance of women's breastfeeding intentions are reflected by the willingness of some mothers to cease taking psychiatric medication to fulfill these wishes.

“*On diazepam at the end of pregnancy for anxiety. Midwife said to come off them before birth. Therefore, low levels plus stopped taking it before birth (1–2 weeks before) so I could breastfeed” (115)*.

The significance of breastfeeding for participants was also reflected by one mother who, despite being otherwise very satisfied about the psychiatric care she received, identified breastfeeding support as the only negative aspect.

“*The nursery staff were excellent and taught me so much. The only lack of support came from my desire to start breastfeeding again, I didn't get any support or encouragement” (101)*.

## Discussion

### Main Findings

This is the first study to investigate infant feeding practices in women with SMI, admitted to acute psychiatric services in the first postpartum year and the factors associated with this. The majority of women had intended to breastfeed and whilst many achieved this in some capacity, only 42.2% reported exclusively breastfeeding. This is significantly less than the breastfeeding initiation rate in the general population which is around 74% (3). However, in women intending to breastfeed, the rate of actual breastfeeding was higher, as was exclusivity, and intentions were a key factor associated with actual method of feeding in this cohort. Antenatal intentions to breastfeed are also strongly predictive of breastfeeding initiation and duration in women without SMI (5, 6).

Fulfilling breastfeeding intentions was central to women and was reflected in free-text comments. Some women described stopping psychotropic medication to fulfill breastfeeding intentions; a significant decision with a myriad of potentially serious implications to mother and infant, as stopping medication can be associated with relapse. The association between unfulfilled breastfeeding intentions and common mental health condition has been documented in previous literature (10) but this is the first study to our knowledge to demonstrate this in women with SMI.

Breastfeeding research in a non-SMI population consistently demonstrates that maternal demographic factors play a significant role in women's attitudes toward breastfeeding and their likelihood of initiating and maintaining breastfeeding (4, 25). Research in women without SMI from high income countries highlights that the highest incidence of breastfeeding is among mothers who are 30 and over, from minority ethnic groups, with post-secondary school education, in managerial and professional occupations, whilst poverty and low socio-economic status are associated with lower odds of breastfeeding (4, 5). This was mirrored in our analysis and irrespective of infant feeding intention, non-Caucasian ethnicity was significantly associated with breastfeeding, as was employment status. A possible explanation could be that unemployed women may be unable to work due to their mental illness and the severity of their illness may also impact their ability to breastfeed. This, together with cultural differences in attitudes toward breastfeeding, family and social support require specific investigation in women with SMI.

There is also evidence to suggest that overweight (25–29.9 kg/m2) and obese (≥30 kg/m2) women (without SMI) are less likely to initiate and continue breastfeeding (26). Our analysis found similar results, with overweight women in the cohort having lower odds of breastfeeding. As women with SMI have an increased risk of high body mass index (BMI) due to inadvertent weight gain associated with certain psychotropic medications, this is a particularly important finding. As with women without SMI, it is important for midwives and other maternity care providers to understand and recognize factors which influence women's decisions about infant feeding, in order to individualize the support they provide.

A key finding from our analysis relates to inconsistent and confusing advice from health care providers against breastfeeding. Over a quarter were advised to stop breastfeeding because of psychotropic medications they were taking, despite very few reporting medications usually avoided during breastfeeding and lactation. For many women, insufficient or contradictory information enhanced their distress and unmet needs, leading to a lack of confidence in healthcare providers and disregard for medical advice, or over-reliance on their own research. Findings from this study mirror those from a previous review into breastfeeding and (non-psychotropic) medication use in women without SMI, that “the decision-making process between health professionals and women is usually not a negotiated process, and women are often asked to stop breastfeeding whilst taking a medicine. Women, in turn, are left dissatisfied with the advice received, many choosing not to initiate therapy or not to continue breastfeeding”(30).

The breastfeeding vs. medication dilemma has been well-established (31), however the unique implications for SMI and psychotropic medicine require further attention. Further training and resources specific to infant feeding in the context of SMI is required across the different healthcare services involved in the care of women with perinatal SMI to ensure consistency of information and support provided. Health professionals must also recognize the importance of women's infant feeding intentions and help to manage their expectations of breastfeeding, supporting them to make decisions about infant feeding which balance the risks of exposure to medication, with the risks of untreated SMI.

The findings from this study demonstrate that breastfeeding can be a primary consideration for women with SMI, yet this is not reflected in current service provision. In particular, women require clearer information and improved support regarding the use of medication whilst breastfeeding throughout pregnancy and the postnatal period. The impact of the COVID-19 pandemic on breastfeeding outcomes in women with mental illness is not yet clear. Evidence suggests the pandemic may have worsened the mental health of women in the early postpartum period (32) and the reduction of dedicated breastfeeding support could be detrimental to mother and infant (33). It is imperative that further research is conducted to understand how to best support women with SMI and their infant feeding needs.

### Limitations

This study was a secondary data analysis of a cohort of women with perinatal SMI and qualitative data was derived from free-text questionnaire responses, rather than interviews, we therefore acknowledged that the depth of analysis may be more limited (34).

Response rate to questions about infant feeding intentions and actual methods in the sample was good but data regarding breastfeeding initiation and duration, or details of mix feeding (i.e., expressed breast milk and formula or baby put to breast and how often) were not collected. Attitudes toward breastfeeding or how they had fed previous children (if applicable) were also not collected, and the construct of infant feeding self-efficacy was not considered. Attitudes toward breastfeeding, previous experience and self-efficacy are all recognized factors affecting breastfeeding initiation and continuation in women without SMI (26) and could be Key to explaining the associations with infant feeding outcomes in this population. These potential predictors should be explored further in future studies.

In addition, data was retrospectively self-reported by Participants 1-month following discharge from psychiatric services, meaning initial experiences of infant feeding could have occurred over a year prior, introducing the risk of recall bias. Retrospective collection of infant feeding measures also means psychiatric illness could have occurred after breastfeeding began and therefore, it is unclear if SMI contributed to breastfeeding outcomes or if breastfeeding led to the mental illness. Responses may also have been subject to social desirability bias as participants may have wanted to answer with what they felt was the “better” method of feeding. We also acknowledge the use of multiple testing and the probability of finding associations that are not in the data.

### Implications

Almost a quarter of the sample formula fed despite wanting to breastfeed and inconsistent advice relating to breastfeeding and psychotropic medication indicates that further training is required for maternity and mental health care providers, about the risks and benefits of breastfeeding for women with SMI, and how to tailor infant feeding advice to women at risk of a postpartum psychiatric illness. Future research should focus on other potential predictors of breastfeeding in this populations, as well as women's experiences of infant feeding and interventions which tailor infant feeding support to women with mental illness.

## Conclusion

This study is the first, to our knowledge to explore the infant feeding outcomes of women admitted to psychiatric services in the first postpartum year. The findings demonstrate that women with perinatal SMI intend to breastfeed and for the majority, this intention is fulfilled. However, compared with women without SMI, the rate of exclusive breastfeeding was lower in this cohort, and duration of breastfeeding in this population remains unclear.

## Data Availability Statement

The raw data supporting the conclusions of this article will be made available by the authors, without undue reservation.

## Ethics Statement

The studies involving human participants were reviewed and approved by NHS Research Ethics Committee, London-Camber well St. Giles committee (number: 14/LO/0765) and this secondary data analysis was conducted in accordance with the Declaration of Helsinki and General Data Protection Regulations. The patients/participants provided their written informed consent to participate in this study. Written informed consent was obtained from the individual's for the publication of any potentially identifiable images or data included in this article.

## Author Contributions

KT and LH contributed to conception and design of the study. SJ and KT dealt with data collection. LP organized the database and had an overview of statistical analysis. NB and SJ analyzed and interpreted the data. NB drafted the manuscript. SJ, LP, and LH wrote sections of the manuscript and contributed to manuscript revisions. All authors contributed to the article and approved the submitted version.

## Conflict of Interest

The authors declare that the research was conducted in the absence of any commercial or financial relationships that could be construed as a potential conflict of interest.
