# Supplementary material for: Factors Affecting Infant Feeding Practices Among Women With Severe Mental Illness
Source: Front Glob Womens Health. 2021 Apr 9;2:624485. doi: 10.3389/fgwh.2021.624485 (PMC8593974; doi:10.3389/fgwh.2021.624485)
Supplement: Supplementary file 1 [file Table_1.DOCX]

| **Appendix A, Table 4: Sensitivity analysis including imputed body mass index (BMI) and household income** | | | | | |
| --- | --- | --- | --- | --- | --- |
|  | **Complete case analysis of cohort (n=188)** | | **Women with antenatal intention to breastfeed (N= 163)** | | |
|  |  | |  | | |
| **Variable** | **AOR (95% CI)** | **P value** | **AOR (95% CI)** | **P value** | |
| **Relationship Status (pregnancy)** |  |  |  |  | |
| Partner/Married | 3.4 (0.8-15.3) | 0.11 | 4.4 (0.6-34.5) | 0.16 | |
| **Gestation at birth** |  |  |  |  | |
| Preterm (32-37 weeks) | 0.6 (0.2-1.6) | 0.30 | 0.7 (0.2-2.6) | 0.61 | |
| **Pregnancy planning** |  |  |  |  | |
| Unplanned | 0.7 (0.3-1.6) | 0.39 | 0.7 (0.2-1.8) | 0.42 | |
| **Mode of birth** |  |  |  |  | |
| Assisted vaginal | 0.9 (0.3-2.6) | 0.86 | 0.9 (0.3-3.2) | 0.91 | |
| Caesarean section | 2.2 (0.9-5.5) | 0.07 | 3.0 (0.9-9.8) | 0.07 | |
| **Psychiatric Onset** |  |  |  |  | |
| Pregnancy | 1.8 (0.6-5.5) | 0.30 | 1.6 (0.4-6.5) | 0.49 | |
| Postpartum | 1.5 (0.5-4.2) | 0.42 | 2.0 (0.5-8.0) | 0.32 | |
| **Psyc Diagnosis (for index admission)** |  |  |  |  | |
| Bipolar Disorder | 0.6 (0.2-1.6) | 0.19 | 0.4 (0.1-1.1) | 0.07 | |
| Schizophrenia | 1.1 (0.2-5.7) | 0.91 | 5.8 (0.4-78.3) | 0.19 | |
| Anxiety disorders | 0.9 (0.3-2.5) | 0.78 | 1.0 (0.3-4.0) | 0.99 | |
| Disorders associated with the puerperium (not elsewhere classified) | 0.5 (0.1-2.3) | 0.38 | 0.3 (0.04-1.6) | 0.15 | |
| Personality Disorder | 0.3 (0.09-1.3) | 0.12 | 0.7 (0.1-4.7) | 0.72 | |
| **Ethnicity** |  |  |  |  | |
| Non-Caucasian | 3.6 (1.3-9.7) | 0.01 | 6.1 (1.5-24.7) | 0.01 | |
| **Age** |  |  |  |  | |
| 25-34 | 2.3 (0.8-6.6) | 0.13 | 3.8 (1.0-14.0) | 0.05 | |
| 35-49 | 0.9 (0.3-2.9) | 0.83 | 1.3 (0.3-5.9) | 0.73 | |
| **Education** |  |  |  |  | |
| Post-secondary education | 0.4 (0.1-1.2) | 0.07 | 0.1 (0.03-0.8) | 0.02 | |
| Degree and above | 0.6 (0.1-3.0) | 0.55 | 0.2 (0.02-1.7) | 0.13 | |
| **Employment prior to pregnancy** |  |  |  |  | |
| Working | 2.6 (1.1-6.3) | 0.03 | 2.6 (0.8-8.4) | 0.11 | |
| **Smoking status (during pregnancy)** |  |  |  |  | |
| Smoker | 0.8 (0.3-2.3) | 0.59 | 0.5 (0.1-1.5) | 0.20 | |
| **BMI** |  |  |  |  | |
| Overweight | 0.5 (0.2-1.2) | 0.12 | 0.2 (0.08-0.8) | 0.02 | |
| Obese | 0.7 (0.3-1.8) | 0.49 | 0.5 (0.1-1.6) | 0.07 | |
| **Household Income** |  |  |  |  | |
| Middle | 0.9 (0.4-2.4) | 0.90 | 0.9 (0.3-2.7) | 0.83 | |
| High | 2.3 (0.7-7.6) | 0.15 | 4.0 (0.9-17.9) | 0.07 | |
